# Explainable artificial intelligence (XAI) in schizophrenia: enhancing transparency in clinical decision-making

**DOI:** 10.3389/fsysb.2026.1898853

**Published:** 2026-09-28

**Authors:** Kshitij Srivastava, Ruby Srivastava

**Affiliations:** 1 Department of Psychiatry, Ganesh Shankar Vidyarthi Memorial (GSVM) Medical College, Kanpur, Uttar Pradesh, India; 2 Department of Chemistry, Indian Institute of Technology Bombay, Mumbai, India

**Keywords:** artificial intelligence, explainable artificial intelligence, machine learning, magnetic resonance imaging, schizophrenia

## Abstract

Schizophrenia is a chronic and severe psychiatric disorder affecting nearly 1% of the global population, characterized by positive symptoms such as hallucinations and delusions, negative symptoms including avolition and affective flattening, and cognitive impairments that compromise social and occupational functioning. Despite advances in psychiatry, diagnosis remains largely symptom-based, relying on criteria from the Diagnostic and Statistical Manual of Mental Disorders (DSM-5) and the International Classification of Diseases (ICD-11), highlighting the need for objective biomarkers and improved clinical decision support. Neuroimaging modalities, including structural and functional magnetic resonance imaging (MRI/fMRI), diffusion tensor imaging (DTI), positron emission tomography (PET), as well as electrophysiological techniques such as electroencephalography (EEG) and magnetoencephalography (MEG), have revealed abnormalities in brain connectivity, dopaminergic signaling, sensory processing, and network dysfunction associated with schizophrenia. Recent advances in artificial intelligence (AI), particularly machine learning (ML), have enabled prediction of diagnosis, treatment response, and relapse risk using multimodal clinical, neuroimaging, and genetic data. However, the “black-box” nature of many AI models limits transparency, interpretability, and clinician trust, restricting translation into practice. Explainable artificial intelligence (XAI) addresses these challenges by providing interpretable, transparent, and clinically accountable models that support decision-making while preserving ethical and regulatory standards. By integrating explainable models with multimodal data, clinicians can use them as an adjunct for precision psychiatry, facilitating biomarker discovery, personalized treatment strategies, and trustworthy, actionable decisions, ultimately enhancing transparency and improving outcomes in schizophrenia care.

## 1 Introduction

Schizophrenia is a mental disorder that belongs to a group of illnesses called psychotic disorders. It is characterized by symptoms such as hallucinations, delusions, lack of motivation, reduced emotional expression, disorganized thinking, and problems with memory and thinking abilities. It usually emerges in late adolescence or early adulthood and is associated with major social and occupational dysfunction. The lifetime morbid risk is commonly estimated around 0.7%–1%, and the disorder is associated with reduced life expectancy, high unemployment, and substantial suicide risk (McGrath et al., 2008; McGrath and Susser, 2009). The World Health Organization (WHO) estimates that approximately 20 million people worldwide are affected by schizophrenia (Mayo Clinic, 2020). Global burden analyses continue to show schizophrenia as a major source of years lived with disability despite its relatively low prevalence (Cohen et al., 2022). Clinically, schizophrenia is still diagnosed through structured psychiatric assessment, longitudinal history, mental-state examination, collateral information, and the exclusion of substance-induced, neurological, medical, and affective causes of psychosis (Saha et al., 2005; 2006; McGrath et al., 2004). Current treatment standards are comparatively well established. The APA guideline recommends a documented, person-centered treatment plan with antipsychotic medication and monitoring for effectiveness and adverse effects, continuation of antipsychotic treatment after response, clozapine for treatment-resistant schizophrenia or substantial suicide risk (Brådvik, 2018; Sher and Kahn, 2019), and long-acting injectable antipsychotics for patients who prefer them. National Institute for Health and Care Excellence (NICE) recommends oral antipsychotic medication together with family intervention and individual Cognitive behavioral therapy (CBT) for first-episode psychosis, early-intervention services, family support, and cardiovascular and metabolic monitoring. NICE also recommends clozapine after inadequate response to two adequate antipsychotic trials, one of which should be a non-clozapine second-generation agent, and suggests depot or long-acting injectable treatment when preference or covert nonadherence makes this clinically important. Recent international consensus guidance has tried to make these pharmacologic sequences more algorithmic and globally usable (Cruz Rivera et al., 2020). These background facts matter because they define the benchmark any AI system must beat or at least complement. In schizophrenia, that benchmark is not a blood test or radiology readout (Burchell et al., 2023). It is longitudinal, inherently multimodal clinical reasoning under uncertainty. That is why the most clinically plausible AI tools are those that help clinicians synthesize complexity, personalize intervention intensity, or detect deterioration earlier than periodic clinic visits can (Cui et al., 2021).

Although the precise etiology remains unclear, it is widely accepted that schizophrenia arises from a complex interplay of genetic, environmental, neurobiological, neurodevelopmental, and immunological factors. Symptom severity and progression vary over time, often involving periods of exacerbation and remission. Typically, onset occurs in the early to mid-20s in men and late 20s in women, while cases in children are rare and prevalence increases with age (Pompili et al., 2007). According to the DSM-5, a diagnosis of schizophrenia requires the presence of at least two core symptoms for a minimum duration of 1 month, along with significant impairment in social or occupational functioning (Professional, C. C. M., 2023a; Professional, C. C. M., 2023b).

Treatment approaches typically include antipsychotic medications, adjunct therapies based on symptom profiles, psychotherapy such as CBT, and in certain cases, electroconvulsive therapy (ECT). The diagnosis of schizophrenia currently relies heavily on the subjective assessment of behavioral symptoms by clinicians, including DSM-5 to guide the evaluations; however, clinical judgment and practitioner experience remain central to the final diagnosis. Consequently, the diagnostic process is often vulnerable to bias, inconsistency, and misclassification, particularly in early-stage or atypical presentations. The lack of a universally accepted, objective diagnostic test for schizophrenia continues to hinder timely and accurate identification of the disorder. Therefore, there is a growing need for biological data-driven approaches that are automated, reproducible, and reliable, and that can be applied across diverse clinical settings without requiring highly specialized expertise.

Despite extensive research, the underlying pathophysiology of schizophrenia remains poorly understood, with only a limited number of consistent biological findings reported (Zhou et al., 2022). At present, objective and quantifiable diagnostic biomarkers are lacking (Torous et al., 2021), and diagnoses based primarily on observable symptoms demonstrate variable reliability. Furthermore, schizophrenia exhibits significant clinical heterogeneity, with overlapping symptoms across other psychiatric conditions, raising concerns regarding the validity of defining it as a single disease entity (Perrin, 2015). Neuroimaging techniques, such as functional magnetic resonance imaging (fMRI), have shown promise but are not yet sufficient for standalone diagnosis. The diagnostic and prognostic studies related to schizophrenia is given in Table 1.

**TABLE 1:** Representative diagnostic and prognostic studies related to Schizophrenia. SSD (schizophrenia spectrum disorders); ARMS (at-risk mental state); ERP (event-related potential); VBM (voxel-based morphometry).

Study	Sample	Data type	AI method	Reported performance	Main limitation
Cui et al., 2022a	662 patients with schizophrenia; 613 controls	Multisite structural MRI	Deep neural networks using VBM-informed features	81.25% correctly classified patients across eight site-validation experiments	Case-control design, healthy controls as comparator, not a real-world diagnostic test
Zhang et al., 2023	Sample size not stated in retrieved abstract	T1 structural MRI	3D deep learning on whole-brain structure	AUC 0.99 on unseen structural MRI scans	Very high internal performance raises concern for dataset specificity and limited transportability
Weng et al., 2024a	Nine global centers	Structural MRI	3D ResNet, leave-one-center-out validation	AUC 0.82 across international datasets	Better generalizability than typical single-site studies, but still largely case-control
Hu et al., 2021a	1,010 total; 508 schizophrenia, 502 controls	Structural MRI plus polygenic risk score	SVM, logistic regression, ensemble learning	Fusion model improved multisite classification stability; exact metric not stated in retrieved abstract	Biomarker fusion promising, but performance details and clinical transportability remain limited
Sasabayashi et al., 2021	65 ARMS participants; 17 transitioned to psychosis	Clinical, cognition, sMRI, ERPs, fatty acids	Linear SVM multimodal prediction	Predicted psychosis onset; exact metric not stated in retrieved abstract	Small high-risk cohort, transition endpoint still distal from confirmed schizophrenia
Hansen et al., 2025	Routine EHR cohort; exact sample not stated in retrieved abstract	Structured EHR data plus clinical notes	Machine learning on routine clinical data	Schizophrenia progression was easier to predict than bipolar disorder; clinical notes were particularly informative	Real-world setting is a strength, but metric details were not available in the retrieved abstract
Di Camillo et al., 2024	Meta-analysis: 119 studies; 12,723 SSD, 13,196 controls	MRI studies	Meta-analysis of multivariate MRI classification	Pooled sensitivity 79.1%; specificity 80.0%	Literature heterogeneity and predominance of case-control imaging studies

The values in the table are summarized from the cited primary studies and meta-analysis. *(Gumley et al. 2022).

Recent advancements in AI, particularly in Natural Language Processing (NLP) and ML, have opened new avenues for developing cost-effective and less intrusive diagnostic tools. These technologies enable large-scale analysis of clinically relevant data, including Electronic Health Records (EHRs) and patient-generated content such as speech and text. Social media platforms, in particular, have emerged as valuable data sources, as individuals frequently share personal experiences (Fuchs, 2015), emotions, and behavioral patterns online (Zarrinkalam et al., 2018; Saha et al., 2022; Prieto et al., 2014). This has led to the development of a novel research domain known as Mental Illness Detection and Analysis on social media (MIDAS). Early MIDAS studies primarily utilized linguistic and statistical features, such as Linguistic Inquiry and Word Count (LIWC), term frequency–inverse document frequency (TF-IDF), and n-grams, in combination with traditional ML algorithms including Support Vector Machines (SVM), Adaptive Boosting (AdaBoost), and Decision Trees. These approaches have been applied to detect a range of mental health conditions, including post-traumatic stress disorder, depression, bipolar disorder, seasonal affective disorder, and schizophrenia (Coppersmith et al., 2014; Mitchell et al., 2015).

However, the application of such techniques in schizophrenia, is still evolving. While recent AI models have demonstrated high predictive accuracy, they often function as “black-box” systems, offering limited interpretability. This lack of transparency is a critical concern in healthcare applications, where understanding the rationale behind decisions is essential. Consequently, there is an increasing emphasis on XAI to enhance model interpretability and trustworthiness in psychiatric diagnosis. The primary objective of current research is to systematically evaluate XAI approaches for the automated detection of schizophrenia using XAI approaches to achieve an optimal balance between predictive performance and interpretability, ensuring that AI-driven systems can effectively support clinical decision-making while remaining transparent and reliable (Kerz et al., 2023).

## 2 Search strategy and evidence categorization

A systematic literature search was conducted in PubMed/MEDLINE, Scopus, Web of Science Core Collection, PsycNet, bioRxiv, medRxiv in accordance with the PRISMA 2020 guidelines. The search covered studies published from 1 January 2015 to 29 June 2026 and combined controlled vocabulary (MeSH terms) with free-text keywords related to schizophrenia spectrum disorders, artificial intelligence, machine learning, deep learning, and explainable artificial intelligence, including specific interpretability methods such as SHAP, LIME, Grad-CAM, Integrated Gradients, and attention mechanisms. Filters were used for only peer-reviewed articles published in English involving human subjects in Medicine, Computer Science and Neuroscience. In addition, manual searches of reference lists and forward citation tracking were performed to identify any additional eligible studies.

Search strings (“Schizophrenia” [Mesh] OR schizophrenia OR “schizophrenia spectrum disorder*” OR psychosis OR psychotic disorder*) AND (“Artificial Intelligence” [Mesh]OR “Machine Learning” [Mesh]OR “Deep Learning”OR “Artificial Intelligence”OR “Machine Learning”OR “Deep Learning”OR “Neural Network*”OR CNN OR RNN OR Transformer* OR XGBoost OR Random Forest OR Support Vector Machine OR SVM) AND (“Explainable Artificial Intelligence” OR “Explainable AI”OR XAI OR Explainability OR Interpretability OR “Interpretable Machine Learning” OR SHAP OR “Shapley Additive Explanations” OR LIME OR “Local Interpretable Model-agnostic Explanations” OR Grad-CAM OR “Integrated Gradients” OR “Attention Mechanism” OR “Counterfactual Explanation*” OR Saliency) is used accordingly to the database and repositories search panels.

Studies were included if they (i) involved individuals with schizophrenia spectrum disorders or populations at clinical high risk for psychosis; (ii) applied AI, ML, deep learning (DL), or related computational approaches integrated with explainability or interpretability techniques (e.g., SHAP, LIME, Grad-CAM, Integrated Gradients, attention mechanisms, or counterfactual explanations); (iii) reported applications related to diagnosis, prognosis, treatment response prediction, relapse prediction, biomarker discovery, digital phenotyping, or clinical decision support; (iv) were original, peer-reviewed research articles published in English; and (v) provided sufficient methodological details regarding the AI model and explainability framework. Studies were excluded if they lacked an explainability component, focused on disorders other than schizophrenia without subgroup analyses, were editorials, conference abstracts, case reports, book chapters, dissertations, or non-human studies, or if the full text was unavailable.

To facilitate structured synthesis, the selected studies were categorized into diagnostic applications, neuroimaging-based predictive models, treatment-response prediction systems, multimodal biomarker integration studies, and conceptual or future-oriented perspectives. Methodological quality assessment and risk-of-bias evaluation were conducted by examining model transparency, reproducibility, external validation, dataset representativeness, and potential conflicts of interest. This rigorous methodology ensured a robust, reliable, and thematically coherent body of evidence, thereby strengthening the validity and integrity of the review findings. The literature selection process followed the principles of the PRISMA 2020 guidelines to ensure transparent identification, screening, eligibility assessment, and inclusion of relevant studies (Page et al., 2021; Haddaway et al., 2022). See Figure 1.

**FIGURE 1:**
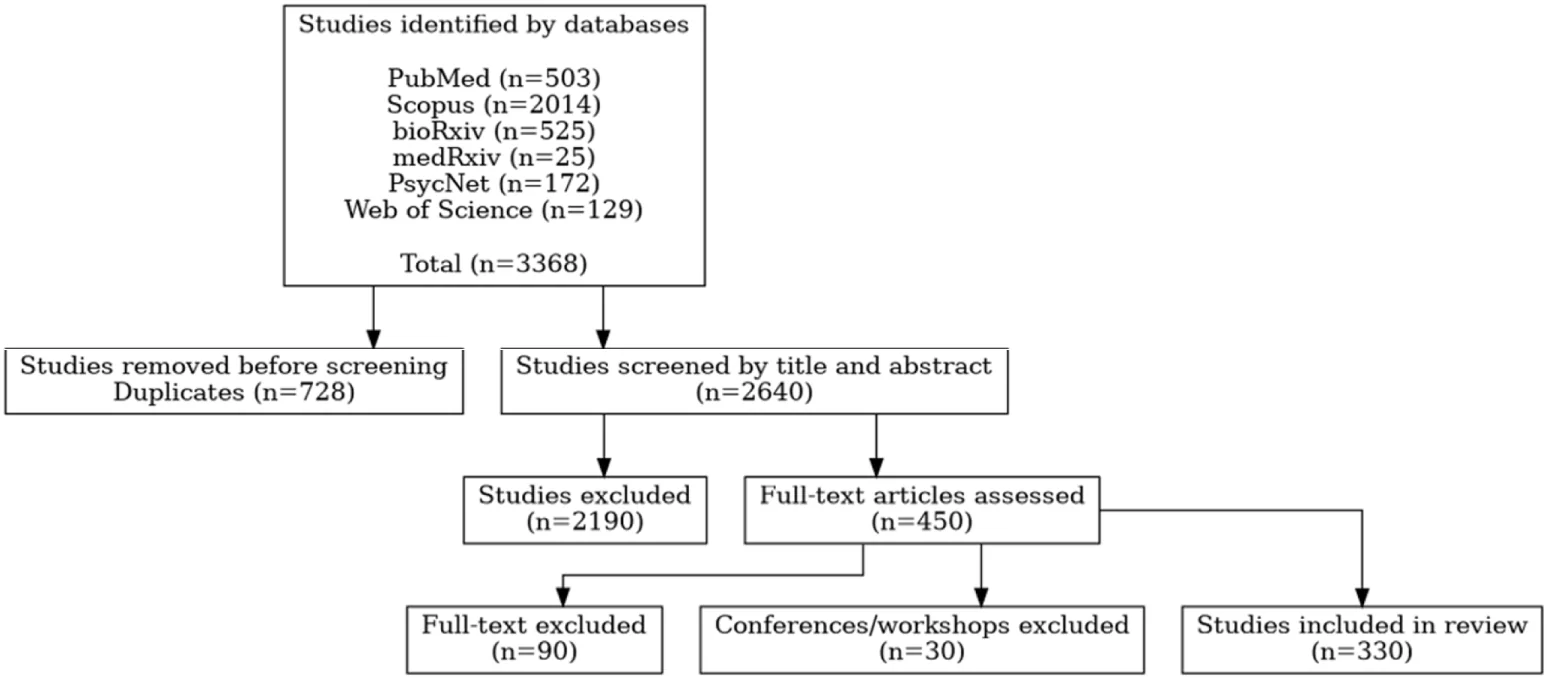
PRISMA 2020 flow diagram illustrating the identification, screening, eligibility assessment, and inclusion of studies related to XAI applications in schizophrenia. The literature selection process followed predefined inclusion and exclusion criteria to ensure methodological rigor, transparency, and clinical relevance. Total 330 studies were selected in this study.

Several factors may have influenced the comprehensiveness and interpretation of the evidence presented in this review. First, although the included studies were critically appraised, a formal standardized methodological quality assessment was not performed because of the substantial heterogeneity in study designs, AI methodologies, imaging modalities, and outcome measures, which limited direct comparisons across studies (Page et al., 2021; Shea et al., 2017). Second, the potential influence of publication and funding bias cannot be excluded, as studies reporting favorable AI performance are more likely to be published than those with negative or inconclusive findings, potentially leading to an overestimation of diagnostic accuracy and clinical utility (Ioannidis, 2005). Third, temporal bias may exist because AI algorithms, computational methods, and explainability techniques evolve rapidly, meaning that earlier studies may no longer reflect the current state of the field. Geographic bias is also evident, as many studies originate from a limited number of countries and academic centers, reducing the generalizability of findings to underrepresented populations and healthcare systems (WHO, 2025). Finally, patient perspectives remain largely absent from the existing literature. Few studies have investigated patient acceptance, understanding, trust, privacy concerns, or preferences regarding AI-assisted psychiatric care, despite these factors being essential for successful implementation and shared clinical decision-making (Miller, 2019; Ghassemi et al., 2021).

A limitation of this review is also the inclusion of preprint studies as part of the evidence base. Although preprints provide timely access to emerging scientific findings and are particularly valuable in rapidly evolving research areas, they have not yet undergone formal peer review and may therefore be subject to methodological revisions, changes in interpretation, or even withdrawal following the review process. Consequently, some of the findings derived from these studies should be interpreted with appropriate caution. To minimize this limitation, the available evidence was critically evaluated alongside peer-reviewed literature; however, future updates of this review should reassess these conclusions as preprints are formally published or revised.

## 3 Methods

AI, introduced by John McCarthy and conceptually advanced by Alan Turing, has transformed healthcare through automated disease detection, medical imaging analysis, and clinical decision support (Akinrinmade et al., 2023). ML and DL techniques, including CNNs, RNNs, BiLSTM, BERT, RoBERTa, and MentalBERT, have enabled accurate prediction and classification of mental and neurological disorders using neuroimaging, speech, and behavioral data (Akinrinmade et al., 2023). Recent approaches combine interpretable linguistic, emotional, and personality-related features with advanced transformer-based architectures to improve mental health assessment while enhancing explainability (Malik et al., 2019; Mintz and Brodie, 2019; Zhang et al., 2022). These AI-driven methods have shown significant potential in diagnosing and monitoring conditions such as schizophrenia, depression, anxiety, brain tumors, stroke, and neurodegenerative diseases, thereby supporting more precise and personalized clinical decision-making (Corti et al., 2024; Calisto et al., 2023; Houssein et al., 2021). Despite these advances, many ML and DL models are considered “black-box” systems, meaning their decision-making processes are difficult to interpret, which raises concerns about transparency, trust, and regulatory acceptance in high-stakes fields such as medicine a]. To address this challenge, XAI has emerged as an important approach for making AI models more transparent and clinically interpretable (Vargas, et al., 2017; Emam et al., 2023).

XAI focuses on improving the transparency and increasing trust, reliability, and safe clinical implementation in healthcare settings (Bibi et al., 2025; Srivastava and Srivastava, 2026). An interpretable model enables users to understand the internal functioning of the algorithm, whereas explainable models provide understandable justifications for specific predictions or outcomes. Such transparency is particularly important in clinical decision-making, where incorrect predictions may have serious consequences and where regulatory frameworks, including GDPR and clinical decision-support guidelines, emphasize accountability and explainability (Nouis et al., 2025). To enhance clinician confidence and facilitate integration into healthcare workflows, XAI bridges the gap between complex AI systems and human clinical reasoning through methods that reveal influential features and decision pathways. XAI techniques are broadly categorized into model-specific and model-agnostic approaches. Model-specific techniques, including Grad-CAM and Integrated Gradients, are designed for architectures such as deep neural networks and are widely used to identify diagnostically relevant regions in medical imaging (Srivastava and Srivastava, 2026). In contrast, model-agnostic approaches such as SHapley Additive exPlanations (SHAP) and Local Interpretable Model-Agnostic Explanations (LIME) can explain predictions from nearly any machine learning model by quantifying the contribution of individual features, although these methods may require substantial computational resources (Liu et al., 2025).

XAI approaches can also be classified as global or local explanations. Global methods describe the overall behavior and feature importance of a model across the entire dataset, whereas local methods explain individual predictions, such as identifying why a particular patient was categorized as high risk (Kerz et al., 2023). Additionally, XAI includes ante-hoc (intrinsically interpretable) models, such as decision trees, and *post hoc* explanation techniques that interpret complex “black-box” systems after training using approaches like saliency maps, SHAP, or LIME (Peng et al., 2025). In mental health applications, interpretable frameworks such as LIME and AGRAD have been employed to identify meaningful linguistic patterns and word categories associated with disorders including Attention-Deficit/Hyperactivity Disorder (ADHD), anxiety, bipolar disorder, depression, and psychological stress, thereby improving transparency and trust in AI-assisted diagnosis and assessment. XAI methods are also used across different tasks such as classification, regression, segmentation, detection, clustering, and prediction, helping explain AI decisions in many application areas, including medical imaging (Houssein et al., 2022). More recently, XAI approaches have been grouped into four broader categories: feature visualization techniques (such as Grad-CAM), hierarchical and causal interpretability methods (such as Layer-wise Relevance Propagation), self-supervised and federated learning approaches, and dynamic or multimodal interpretability frameworks.

### 3.1 Feature visualization techniques

Feature visualization methods provide intuitive image-based explanations but may have reproducibility limitations, while attribution methods like SHAP offer useful local and global explanations, especially for structured data. Emerging approaches involving federated, self-supervised, and multimodal XAI show promise for privacy-preserving and comprehensive interpretation, although many of these methods are still in early stages of development. Feature visualization techniques help interpret deep learning models in medical imaging by highlighting image regions that influence model predictions. Methods such as Grad-CAM generate class-specific heatmaps that show diagnostically important areas, while Grad-CAM++ improves localization by incorporating higher-order gradient information, making it especially useful when multiple pathological regions overlap (Chen et al., 2024). These methods have been successfully applied in tasks such as brain tumor detection and multiple sclerosis subtype classification, where they improve transparency by visually linking model predictions to clinically relevant regions (Mohamed Musthafa et al., 2024; Zhang et al., 2021). More recent developments extend these ideas to Vision Transformers (ViTs), deformable attention models, and pixel-level explanation methods such as Layer-wise Relevance Propagation (LRP), which can provide even finer interpretability in complex diagnostic tasks (Dong et al., 2022; Park et al., 2022; Liang and Zeng, 2024). However, because saliency methods can sometimes reflect model bias or spurious correlations, careful validation and standardized evaluation remain necessary (Saporta et al., 2022; Halder et al., 2024; Zhang et al., 2023; Zarenia et al., 2025).

### 3.2 Hierarchical interpretability methods

Hierarchical interpretability methods provide deeper insight into how neural networks make decisions by tracing predictions through different layers of computation. Techniques such as Layer-wise Relevance Propagation (LRP) and Deep Taylor Decomposition generate detailed relevance maps that show how individual input features contribute to predictions, offering more structured explanations than conventional saliency methods. These methods have shown value in medical imaging applications, including multiple sclerosis and Schizophrenia classification, where they have identified clinically meaningful brain regions and functional connectivity patterns associated with disease (Eitel et al., 2019). More advanced approaches include counterfactual explanations, which show how small changes in input could alter model predictions and support causal reasoning in clinical decision-making (Schulz et al., 2020; Mothilal et al., 2020) to improve interpretability, although they can be computationally complex and remain challenging to integrate routinely into clinical practice.

### 3.3 Self-supervised and federated learning–based XAI methods

Self-supervised learning (SSL) and federated learning (FL) are increasingly being combined with explainable AI to address challenges such as limited labeled data, privacy protection, and model transparency in medical imaging. SSL enables models to learn robust feature representations from unlabeled data, while FL supports collaborative model training across institutions without sharing raw patient data. When integrated with XAI, these approaches can support scalable and privacy-preserving intelligent healthcare systems. For example, contrastive attention models have improved both medical report generation and interpretability by aligning image features with clinically relevant semantic cues (Chowdhury et al., 2021). Comparative studies have also shown that SSL models can outperform conventional supervised models in cross-dataset generalization tasks (Anton et al., 2022). The explainability within federated and self-supervised frameworks remains at an early stage, and standardized interpretability methods for these systems are still under development.

### 3.4 Dynamic and multimodal analysis

Dynamic and multimodal XAI frameworks combine information from multiple imaging modalities such as MRI, CT, PET, and SPECT to improve diagnostic accuracy and model interpretability. Because no single modality captures all aspects of disease, combining structural and functional information can provide a more comprehensive understanding. For example, MRI can detect structural abnormalities associated with schizophrenia, while SPECT reveals functional changes such as altered cerebral blood flow; together they provide complementary biomarkers for disease characterization. Advanced multimodal fusion methods, such as Multimodal Dynamics and Two-Level Dynamic Adaptive Networks (TDAN), use dynamic attention and adaptive fusion mechanisms to identify the most informative modalities for each patient while preserving interpretability through modality contribution visualization (Han et al., 2022; Huang et al., 2022). However, recent studies suggest that balancing feature-level and modality-level information is crucial for achieving both robust predictions and meaningful explanations (Zhao and Wang, 2024).

XAI methods such as Grad-CAM, SHAP, LIME, Integrated Gradients, and prototype-based networks have been widely used across medical imaging modalities including MRI, CT, PET, and ultrasound to improve the interpretability of AI models by generating saliency maps, feature importance scores, and counterfactual explanations (Holzinger et al., 2019; Ghassemi et al., 2021; Lu and Zhang, 2022). However, many of these are *post hoc* methods, meaning explanations are generated after the model is trained, raising concerns about whether they truly reflect how the model makes decisions. To address this, newer approaches such as self-explainable AI (S-XAI) aim to embed interpretability directly into model training, potentially improving both transparency and performance (Bharati et al., 2024; Lai, 2024). In radiology and nuclear medicine, limited interpretability remains a major barrier to routine clinical adoption of deep learning models (Černevičienė and Kabašinskas, 2024). Evaluating XAI methods is also challenging and often relies on metrics such as localization accuracy (using measures like Dice coefficient or Intersection over Union (IoU) to compare highlighted regions with expert annotations), faithfulness correlation (assessing whether explanations reflect actual model reasoning), and interpretability scores based on clinician feedback. While useful, these metrics have limitations related to subjectivity, dependence on annotations, and incomplete representation of explanation quality. Major challenges remain, including lack of consensus on the best XAI methods for specific clinical tasks, trade-offs between interpretability and performance, limited real-world validation, and increasing complexity introduced by federated learning and newer architectures such as Vision Transformers (ViTs) (Bharati et al., 2024; Lai, 2024).

The AI literature in schizophrenia spans classical supervised machine learning, deep learning, natural language processing, imaging radiomics, and multimodal fusion. Classical machine-learning models such as support vector machines, logistic regression, random forests, gradient boosting, and elastic-net classifiers remain common in smaller structured datasets because they are relatively stable and interpretable. Deep learning is more typical when raw or minimally processed high-dimensional inputs are available, especially structural MRI volumes, resting-state connectivity matrices, multichannel EEG, raw audio, and increasingly multimodal inputs. NLP studies use acoustic, lexical, syntactic, semantic, and graph-theoretic features to quantify thought disorder or psychosis risk, while multimodal models attempt to fuse symptoms, cognition, imaging, polygenic risk scores, and digital-behavioral signals (Cui et al., 2022a). The data types are correspondingly broad. Structural MRI contributes voxel-based morphometry, cortical thickness, subcortical volumes, and radiomic texture features; resting-state or task fMRI contributes functional connectivity and network-level signatures; EEG contributes spectral power, entropy, nonlinear complexity, microstate-related features, and event-related potentials; EHRs contribute diagnoses, medications, service use, laboratory results, and free-text notes; speech and text contribute prosody, pauses, coherence, semantic drift, and linguistic organization; genomics and other omics contribute polygenic risk scores, methylation, and miRNA features. Recent work also incorporates smartphone sensors, wearables, and ecological momentary assessments into relapse-prediction and symptom-forecasting pipelines (Di Camillo et al., 2024).

## 4 Application of XAI in schizophrenia

### 4.1 Neuroimaging

Neuroimaging has become a fundamental tool for understanding the pathophysiology, diagnosis, and treatment monitoring of schizophrenia by revealing abnormalities in brain structure, function, connectivity, and neurochemistry. Structural neuroimaging techniques, including computed tomography (CT) and structural magnetic resonance imaging (sMRI), have consistently demonstrated enlarged ventricles, cortical thinning, reduced gray matter volume, and white matter abnormalities in patients with schizophrenia, particularly within the prefrontal cortex, temporal lobes, hippocampus, thalamus, and basal ganglia regions associated with cognition, memory, and emotional regulation (Farahani et al., 2022; Shenton et al., 2010; Crosson et al., 2010). Longitudinal sMRI studies further indicate progressive structural alterations correlated with disease severity and chronicity, while some evidence suggests that antipsychotic treatment may influence brain volume changes associated with clinical improvement (Backhausen et al., 2022; Perlini et al., 2012; Yang et al., 2021; Emsley et al., 2023). Advanced diffusion imaging methods, including diffusion-weighted imaging (DWI), DTI, and DKI, have identified disrupted white matter integrity in major tracts such as the corpus callosum, cingulum bundle, and frontotemporal fasciculi, supporting the dysconnectivity hypothesis of schizophrenia (Varaprasad and Goel, 2025). These abnormalities are thought to arise from impaired synaptic pruning, neuroinflammation, glial dysfunction, and altered neurodevelopmental mechanisms influenced by genetic and environmental factors, including complement component 4 (C4) gene variations (Amador-Tejada et al., 2025; Lee et al., 2013).

Functional neuroimaging approaches, particularly functional magnetic resonance imaging (fMRI), have provided significant insights into altered neural activity and connectivity in schizophrenia. Both resting-state fMRI (rs-fMRI) and task-based fMRI (T-fMRI) measure blood oxygen level-dependent (BOLD) signals to evaluate neural activation and large-scale brain network interactions (Lee et al., 2013). Functional imaging studies have consistently reported disruptions in the default mode network (DMN), fronto-parietal circuits, dorsolateral prefrontal cortex (DLPFC), anterior cingulate cortex (ACC), hippocampus, thalamus, and basal ganglia, which are strongly associated with impairments in working memory, executive functioning, emotional processing, and decision-making (Breiman, 1996). Resting-state studies demonstrated widespread abnormalities in intrinsic connectivity across cognitive and sensory networks, while task-based studies revealed altered activation patterns during working memory, emotional recognition, and cognitive control tasks. Advanced analytical techniques such as Independent Component Analysis (ICA) and Vision Transformer (ViT)-based architectures have enabled unbiased whole-brain analysis, improving identification of novel biomarkers and large-scale connectivity disruptions (Sharifani and Amini, 2023; Górriz et al., 2023; Bishop and Nasrabadi, 2006; Dietterich, 2000; Schölkopf et al., 2002). Furthermore, studies integrating structural and functional imaging have shown strong relationships between morphological abnormalities and altered functional connectivity, emphasizing the widespread network dysfunction underlying schizophrenia.

Molecular and functional imaging modalities such as PET and SPECT further contribute to schizophrenia research by evaluating neurotransmitter systems, glucose metabolism, cerebral blood flow, and neuroinflammatory processes. PET and SPECT are particularly valuable for investigating dopaminergic dysfunction and early biological changes that may precede structural abnormalities detectable by MRI. When combined with CT or MRI, these modalities provide complementary anatomical and functional information, improving diagnostic precision and individualized treatment planning (Bishop and Nasrabadi, 2006; Dietterich, 2000; Schölkopf et al., 2002; Gao et al., 2015; Breiman, 1996). EEG and MEG additionally offer high temporal resolution for monitoring abnormal neural oscillations and electrophysiological disturbances associated with schizophrenia.

Zheng et al. (2021) applied a VGG16-based CNN with transfer learning to fMRI data and achieved classification accuracy of 84.3%, while Chen et al. (2020) combined recursive feature elimination with SVMs to achieve accuracies exceeding 85% (Chen et al., 2020). Multimodal deep learning frameworks integrating sMRI, fMRI, and genomic data have further improved predictive performance. Similarly, Ahmad et al. (2023) utilized resting-state fMRI with Lasso regression and SVM classifiers to achieve 94% accuracy. Recent XAI approaches incorporating Grad-CAM and attention mechanisms have enabled visualization of clinically relevant brain regions contributing to classification decisions, particularly within the frontal cortex and superior frontal gyrus. Though many earlier neuroimaging studies predominantly included medicated patients, potentially confounding imaging findings due to antipsychotic effects (Lesh et al., 2015). Additionally, smaller datasets often report higher classification accuracies but lack generalizability due to clinical heterogeneity and sampling bias (Schnack and Kahn, 2016). Brain parcellation strategies also significantly influence feature extraction and predictive modeling, with anatomical, cytoarchitectural, connectivity-based, and data-driven approaches each offering distinct advantages and limitations (Zilles and Amunts, 2009; Eickhoff et al., 2008; Desikan et al., 2006; Damoiseaux and Greicius, 2009; Roca et al., 2010; Yao et al., 2015; Thirion et al., 2014). Ensemble frameworks such as EMPaSchiz have integrated multiple parcellation schemes using rs-fMRI data from antipsychotic-naïve patients, achieving classification accuracy of 87% while maintaining clinical relevance and scalability (Kalmady et al., 2019).

### 4.2 Radiopharmaceuticals

For neuroimaging applications, PET and SPECT radiotracers must be capable of crossing the blood–brain barrier (BBB) to effectively target brain tissues and visualize neurobiological processes associated with psychiatric disorders (Janevik and Delcev-Stip, 2015). Advances in radiotracer neurochemistry have enabled the development of highly specific and high-affinity probes for investigating molecular pathways implicated in schizophrenia, including neurotransmitter dysfunction, alterations in regional cerebral blood flow (rCBF), and neuroinflammatory processes (Lu and Yuan, 2015; Cumming et al., 2021). Over the past several decades, PET and SPECT imaging have substantially contributed to identifying molecular biomarkers of schizophrenia through non-invasive assessment of functional and neurochemical abnormalities. Common SPECT radionuclides include technetium-99m (99mTc) and iodine-123 (123I), whereas PET commonly utilizes radionuclides such as 18F, 11C, 13N, and 15O for high-resolution brain imaging studies (Brandt et al., 2018; Shukla and Kumar, 2006). PET offers superior spatial resolution, sensitivity, and contrast, making it particularly valuable for metabolic and molecular imaging, while SPECT remains more cost-effective, accessible, and clinically practical, especially when integrated with CT for improved anatomical localization (Saha et al., 1994; Chareonthaitawee et al., 2006).

Molecular imaging studies using PET and SPECT have provided important insights into neurotransmitter dysregulation in schizophrenia, particularly involving dopaminergic, GABAergic, and glutamatergic systems. Dopaminergic dysfunction remains one of the most extensively investigated mechanisms, with PET studies demonstrating elevated dopamine synthesis capacity and increased presynaptic dopamine release, supporting the dopamine hypothesis of schizophrenia. SPECT radiotracers such as [123I] IBZM and [123I] epidepride have been widely used to evaluate D2/D3 receptor activity, extrastriatal dopamine signaling, cognition, and treatment response (Brandt et al., 2018; Shukla and Kumar, 2006). Beyond dopamine, SPECT imaging with [123I] iomazenil has provided evidence of altered GABAA receptor binding and inhibitory dysfunction, although findings remain somewhat variable across studies (Busatto et al., 1994; Kung et al., 1997; Hsiao et al., 2003). Additionally, glutamatergic abnormalities involving NMDA receptor dysfunction have been investigated using tracers such as [123I] CNS-1261, suggesting potential associations with symptom severity and antipsychotic treatment effects (Verhoeff et al., 1999; Pifarré et al., 2010).

### 4.3 EEG sensor data

EEG has emerged as a valuable non-invasive tool for schizophrenia diagnosis due to its portability, high temporal resolution, and ability to capture abnormal neural activity patterns associated with the disorder (Akbari et al., 2021; Khaleghi et al., 2019; Xiao et al., 2022; Campos-Ugaz et al., 2023; Mohammadi et al., 2023). The integration of AI with ML/DL and EEG has significantly improved automated schizophrenia detection by enabling robust feature extraction, classification, and interpretability (Barros et al., 2021; Afzali et al., 2023; Oh J et al., 2019). Traditional ML approaches using wavelet decomposition, nonlinear feature extraction, entropy analysis, and connectivity measures combined with classifiers such as Support Vector Machines (SVM), K-Nearest Neighbors (KNN), AdaBoost, Gradient Boosting, and XGBoost have achieved high diagnostic accuracies ranging from approximately 80%–99% (Alavi et al., 2021; Behrouzian et al., 2023). More advanced DL architectures, including Convolutional Neural Networks (CNNs), Recurrent Neural Networks (RNNs), BiLSTM-GRU, attention-based BiLSTM models, graph convolutional networks, ResNet, and hybrid CNN-LSTM frameworks, have demonstrated superior performance in capturing spatial and temporal EEG dependencies, with several studies reporting accuracies above 95% (Heda et al., 2025; Weng et al., 2024a; Srinivasan and Johnson, 2024; Xu et al., 2024; Lalawat et al., 2025). To improve robustness and clinical applicability, recent studies have incorporated techniques such as Synthetic Minority Over-sampling Technique (SMOTE), feature selection, Generative Adversarial Networks (GANs), and explainable AI (XAI) methods including SHAP, LIME, and Integrated Gradients, enabling identification of critical EEG biomarkers influencing predictions. Additionally, multimodal and connectivity-based approaches integrating coherence, phase-locking value (PLV), and neuroimaging data have further enhanced classification performance and supported the concept of schizophrenia as a dysconnectivity disorder (Yang, 2025). Despite promising results, challenges including limited dataset size, inter-subject variability, computational complexity, lack of cross-site validation, and clinical interpretability remain significant barriers to real-world implementation (Almadhor et al., 2025).

### 4.4 GAIT analysis

Within the framework of the SPECTRA project (Supporting Schizophrenia Patients’ Care with Artificial Intelligence), the primary objective is to integrate multimodal patient data derived from diverse sources, including speech patterns, emotional responses, and locomotion behavior, with a particular emphasis on gait analysis. Advanced deep learning techniques are employed to identify distinctive movement patterns, while XAI methods are utilized to enhance transparency by clarifying the decision-making processes of these models. Using motion capture systems, the research team assessed mental health conditions through standardized clinical scales such as the Positive and Negative Syndrome Scale (PANSS), the Brief Psychiatric Rating Scale (BPRS), and Neurological Soft Signs (NSS). This analysis enabled the identification of 16 quantifiable movement-related markers associated with schizophrenia. To further improve interpretability, *post hoc* XAI techniques, including LIME (Ribeiro et al., 2016) and SHAP (Lundberg and Lee, 2017a), were applied. These methods provide insights into model predictions by highlighting the contribution of individual features. For example, Mishra et al. (Mishra et al., 2024) demonstrated the utility of LIME in identifying significant features in a multi-layer perceptron model used to classify individuals wearing different types of Knee-Ankle-Foot Orthoses. A subset of individuals with schizophrenia exhibits persistent symptoms despite adequate antipsychotic treatment and are categorized as having Treatment-Resistant Schizophrenia (TRS) (Iasevoli et al., 2016). TRS affects approximately 30% of patients with schizophrenia and is often diagnosed at a later stage of the disorder. This delay can hinder timely initiation of more effective treatments, such as clozapine, as well as non-pharmacological interventions, resulting in increased patient burden and economic costs. Therefore, early and accurate identification of TRS is essential for optimizing treatment strategies and improving patients’ quality of life (QoL). Recent studies have explored the use of sensor-based technologies for the detection and monitoring of mental health disorders (Amaro et al., 2023; Cantone et al., 2023; Francese et al., 2021). In this context, the SPECTRA project aims to develop a Decision Support System (DSS) by integrating artificial intelligence techniques with advanced Internet of Things (IoT) technologies. By combining conventional clinical assessments with ICT-based data collection methods and machine learning algorithms, the project seeks to facilitate early diagnosis of TRS. The study involves real patients recruited from the Unit for Treatment-Resistant Psychosis at the University “Federico II” of Naples. Participants are categorized into TRS and non-TRS groups, and both retrospective clinical data (e.g., MRI scans, questionnaire scores, demographic and geographical information) and real-time physiological data (e.g., ECG, EEG, temperature, and audiovisual signals) are collected during clinical evaluations. These heterogeneous datasets are used to train machine learning and deep learning models for early TRS detection. However, a major challenge in clinical adoption of AI systems is the lack of transparency associated with black-box models.

According to Guidotti et al. (2018), black-box models are machine learning algorithms whose internal decision-making processes are not directly understandable by humans, even though they often achieve high predictive accuracy. To address this lack of transparency, the authors distinguish between inherently interpretable (transparent) models and *post hoc* explanation methods. Inherently interpretable models, such as decision trees and linear models, are designed so that their predictions can be understood directly from their structure and parameters. In contrast, *post hoc* explanation approaches aim to explain the behavior of an already trained black-box model without altering its internal architecture. These methods provide either global explanations of the overall model behavior or local explanations for individual predictions, thereby improving transparency while preserving the predictive performance of complex models. Guidotti et al. emphasize that interpretability and explanation are related but distinct concepts, with transparent models offering intrinsic understanding and *post hoc* methods providing approximations of black-box reasoning.

The SPECTRA project incorporates XAI-driven interaction models designed to support clinicians during the diagnostic process by providing interpretable explanations of model outputs (Antoniadi et al., 2021). Additionally, the project aims to develop a comprehensive dataset distinguishing TRS from non-TRS cases, which will contribute to future research in machine learning-based diagnosis of schizophrenia. The study specifically focuses on analyzing gait characteristics in individuals with schizophrenia through visual assessment of walking patterns. The methodology is divided into two primary phases: (i) data acquisition and preprocessing, and (ii) classification with interpretability. Initially, video recordings are obtained from two groups: patients diagnosed with schizophrenia and matched healthy controls using a multi-camera setup. All participants provide informed consent, and collected data are anonymized for inclusion in a publicly accessible dataset.

A body detection algorithm is applied to extract skeletal joint information from the recorded videos, generating 2D joint coordinates for each frame. Although data collection involves a three-camera configuration to ensure comprehensive coverage, a single-camera setup is proposed for real-world implementation due to its practicality. Inspired by state-of-the-art spatial-temporal transformer models such as VideoPose (Pavllo et al., 2019) and MotionBERT (Zhu et al., 2023), a dual-transformer architecture is utilized to reconstruct 3D skeletal representations and perform classification of gait patterns.

To enhance interpretability, the SHAP algorithm is employed to analyze the model’s decision-making process. The SHAP-based visualization, represented as a matrix, illustrates the contribution of different body joints across video frames. In this matrix, columns correspond to frame indices, while rows represent specific joints (e.g., right wrist). The color-coded representation indicates the influence of each feature: white regions denote negligible contribution, red regions highlight features that positively influence the model’s prediction, and blue regions indicate features that negatively impact classification outcomes. This visualization facilitates intuitive understanding of how specific body movements contribute to the model’s predictions, enabling clinicians to focus on critical frames and movement patterns. Consequently, this approach supports a more informed and nuanced interpretation of diagnostic results. Based on prior findings reported by Martin et al. (Martin et al., 2022), several key gait-related features were selected for analysis. These include head posture (defined as the angle between the head and clavicle), number of steps, walking speed, arm sway (evaluated through variations in elbow or armpit angles), presence or absence of facial movements, step length, and variability in step length. These features are essential for assessing gait dynamics, identifying abnormalities, and understanding movement patterns associated with schizophrenia.

### 4.5 Patients hand writing

The disruption in the normal flow and organization of thought, commonly referred to as Formal Thought Disorder (FTD), is widely recognized as a core feature of schizophrenia. FTD has demonstrated prognostic significance, particularly in predicting the transition from first-episode acute transient psychotic disorder or attenuated psychosis syndrome (APS) to schizophrenia (Brucato et al., 2017; Rusaka and Ranc¯ans, 2014). Recent findings indicate that specific FTD-related symptoms such as conceptual disorganization (P2), difficulty in abstract thinking (N5), and unusual thought content (G9) as measured by the PANSS serve as strong predictors of TRS. (Ortiz et al., 2020). At the linguistic level, schizophrenia is characterized by a wide range of abnormalities, including disruptions in semantic cohesion (Mackinley et al., 2021; Willits et al., 2018), reduced syntactic complexity (Çokal et al., 2018), diminished idea density (Moe et al., 2016), and altered linguistic styles such as decreased analytic thinking (Silva et al., 2021). Additional impairments include irregular speech rhythms, particularly in pausing patterns (Çokal et al., 2019a), difficulties in understanding idiomatic expressions (Schettino et al., 2010), challenges in deriving inferred meanings (Pawełczyk et al., 2018), and reduced use of embedded clauses (Çokal et al., 2019b). Advances in automated natural language processing (NLP) techniques offer promising opportunities for systematically analyzing these linguistic features in psychiatric research.

A study aimed to develop and validate a comprehensive clinical screening tool, the 16-item Screening Instrument for Schizophrenic Features in Writing (SISFiW), to capture diverse writing characteristics associated with schizophrenia. The instrument was developed through extensive literature review and expert analysis of 30 writing samples from patients with schizophrenia and subsequently validated in 157 participants, including 114 patients diagnosed according to ICD-10 criteria and 43 healthy controls. Clinical symptoms were assessed using the PANSS and the Scale for the Assessment of Thought, Language, and Communication (TLC), while written samples were evaluated using SISFiW. The findings demonstrated significantly higher SISFiW scores in patients compared with controls [(3.61 ± 1.72) vs (0.49 ± 0.63), p < 0.001], with the tool exhibiting acceptable inter-rater reliability (weighted kappa = 0.72) and internal consistency (Cronbach’s alpha = 0.613). ROC analysis identified an optimal cutoff score of 2, yielding high sensitivity and specificity (93% each), while discriminant analysis highlighted eight key writing features that differentiated patients from controls with classification accuracies of 90.4% for original cases and 88.5% for cross-validated cases, suggesting that SISFiW may serve as a practical complementary measure for formal thought disorder (FTD) assessment. In another related study, the Personalized Advantage Index (PAI) and Bayesian Rule Lists (BRL) methodologies were integrated to identify treatment-responsive subgroups and improve interpretability in schizophrenia treatment prediction. Using baseline demographic and clinical variables, the PAI model predicted week-6 PANSS outcomes, explaining 58% of the variance (R^2^), and classified patients into treatment-indicated (n = 100) and rest-indicated (n = 97) groups using a threshold score of −9.5. This stratification revealed a markedly greater treatment effect in the identified subgroup (Cohen’s d = 1.51) compared with the overall sample (Cohen’s d = 0.82). Subsequently, a BRL model trained using 5-fold cross-validation achieved approximately 74% test accuracy, an AUC of ∼0.76, and an F1 score of ∼0.73, identifying 87 of 197 patients as treatment-indicated, with this subgroup showing a significantly enhanced treatment effect size (Cohen’s d ≈ 1.24; p < 0.0001). The BRL framework generated interpretable if–then decision rules, consistently identifying disturbance of volition and uncooperativeness as major predictive features, while a final model trained on the complete dataset further improved performance with an accuracy of 76%, AUC of 0.81, and F1 score of 0.75, while maintaining stable predictive variables (Peng et al., 2024).

### 4.6 Clinical data

The PAI is a methodological framework used to identify patient subgroups, often referred to as *“treatment-indicated”*, who are more likely to benefit from a specific intervention compared to placebo, thereby supporting personalized medicine approaches (DeRubeis et al., 2014). In addition to guiding individualized treatment strategies, PAI can enhance clinical trial design by improving patient selection and enriching study populations with individuals most likely to respond to treatment. Despite its advantages, a notable limitation of PAI is its lack of interpretability, which makes it challenging for clinicians to understand the underlying decision-making process. Previous studies in depression have successfully utilized PAI to identify predictive variables; however, interpretation of these results remained complex and lacked clearly defined decision thresholds (Webb et al., 2018). To overcome this limitation, an XAI approach known as Bayesian Rule Lists (BRL) was introduced. BRL generates simple, interpretable *if–then* decision rules with explicit cutoffs, thereby improving transparency and clinical usability (Gao et al., 2018; Letham et al., 2015). The combined PAI + BRL framework has previously demonstrated improved identification of treatment-responsive subgroups in depression studies (Martin et al., 2019). A limitation of this combined approach is its reliance on both baseline and post-treatment data, which may restrict its applicability in prospective clinical trials without prior datasets. Nevertheless, it remains valuable for retrospective analysis of failed trials, enabling the identification of responsive subpopulations, and for developing clinical decision-support systems for existing therapies. In a study, the integrated PAI and BRL framework was applied to Schizophrenia using retrospective clinical trial data. The findings indicate that baseline patient characteristics alone can effectively identify subgroups with enhanced treatment response, thereby offering potential improvements in clinical trial outcomes through targeted patient selection. The analysis utilized data from a 6-week randomized, double-blind, placebo-controlled clinical trial (NCT00083668) evaluating extended-release paliperidone for schizophrenia treatment. This trial was successful, and paliperidone subsequently received regulatory approval. De-identified patient-level data were obtained from the YODA Project. Participants were assessed at baseline and randomly assigned to treatment groups. For the current analysis, only the 15 mg/day paliperidone group (n = 113) and the placebo group (n = 120) were included, as the higher dose demonstrated the strongest therapeutic effect. The primary outcome measure was the Positive and Negative Syndrome Scale (PANSS), assessed weekly. After excluding patients with missing baseline data, the final sample consisted of 95 patients in the treatment group and 102 in the placebo group. The PAI model was used to predict week 6 PANSS scores based on baseline clinical and demographic features, explaining approximately 58% of the variance (R^2^). By comparing actual and counterfactual treatment predictions, individualized PAI scores were computed. A threshold value of −9.5 was used to stratify patients into *treatment-indicated* (n = 100) and *rest-indicated* (n = 97) groups. This stratification revealed a significantly stronger treatment effect within the treatment-indicated subgroup (Cohen’s d = 1.51) compared to the overall sample (Cohen’s d = 0.82). Subsequently, these subgroup labels were used to train the BRL model using 5-fold cross-validation, yielding a test performance of approximately 74% accuracy, area under the curve (AUC) of ∼0.76, and F1 score of ∼0.73. Out of 197 patients, 87 were classified as treatment-indicated by the BRL model. This subgroup exhibited an improved treatment effect size (Cohen’s d ≈ 1.24), which was statistically significant across repeated analyses (p < 0.0001). The BRL model generated interpretable if–then rules, consistently identifying clinical features such as high disturbance of volition and uncooperativeness as key predictors of treatment response. These rules demonstrated stability across cross-validation folds and yielded consistent patient classifications. A final BRL model trained on the full dataset achieved 76% accuracy, an AUC of 0.81, and an F1 score of 0.75, while maintaining the same key predictive features (Martin et al., 2019).

## 5 Discussion

### 5.1 Diagnosis and early detection

Diagnosis remains the most heavily studied use case. MRI and fMRI studies dominate the literature, with deep networks and connectome-based methods attempting to extract structural or network signatures of schizophrenia. Multisite work has been important because it forces models to handle scanner effects, demographic heterogeneity, and differences in preprocessing (Hu et al., 2021a). MRI facilitates the identification of structural abnormalities associated with schizophrenia (Shenton et al., 2001). Nevertheless, the high-dimensional and complex nature of MRI data presents substantial challenges for manual interpretation and biomarker extraction by clinicians (Cui et al., 2022b). To address these limitations, deep learning approaches have been increasingly applied to neuroimaging data for schizophrenia detection, owing to their ability to automatically learn hierarchical and non-linear features from high-resolution data (Wang et al., 2022; Sadeghi et al., 2022; Hu et al., 2022). Despite their promise, DL models face notable challenges, particularly the scarcity of large-scale, multisite neuroimaging datasets for robust training and independent validation (Oh S et al., 2019; Oh et al., 2020; Li et al., 2021). This limitation is further compounded by the intrinsic heterogeneity of schizophrenia, which complicates the evaluation of model generalizability across diverse populations (Fang et al., 2022). Consequently, models often exhibit reduced performance when tested on external datasets, hindering their clinical applicability. Although several studies have reported encouraging results using multi-site imaging data (Hu et al., 2021a; Yan et al., 2019; Cui et al., 2022b; Orban et al., 2018; Zeng et al., 2018), many have relied on datasets derived from a single country, thereby neglecting potential variations due to ethnic and demographic differences (Hu et al., 2021b; Yan et al., 2019; Cui et al., 2022b). VBM, a widely used structural MRI technique, enables the assessment of regional brain volume changes across the entire brain (Cannon et al., 2015; Haijma et al., 2013; Gur et al., 2019; Chand et al., 2020; Li et al., 2023). In this context, grey matter volume (GMV) has been identified as a potential biomarker for structural abnormalities associated with schizophrenia (Xie et al., 2022). Additionally, studies have reported significant alterations in white matter (WM) integrity and cerebrospinal fluid (CSF) dynamics in patients with schizophrenia compared to healthy controls (Pérez-Iglesias et al., 2010; Kavcic et al., 2008; Uranova et al., 2001; Kartalcı et al., 2021; Gur et al., 1994; Gur et al., 1991), including reduced white matter integrity and abnormal ventricular volume and CSF flow (Pérez-Iglesias et al., 2010; Kartalcı et al., 2021). To improve model interpretability, several XAI techniques have been proposed, including SHAP and Grad-CAM (Selvaraju et al., 2020; Wang et al., 2020). Among these, SHAP has gained particular prominence due to its ability to provide feature-level contributions for individual predictions, thereby enabling subject-specific interpretations (Xie et al., 2022; Bang et al., 2021; Bllester et al., 2023). Unlike methods that assess features independently, SHAP evaluates the weighted marginal contributions of all features, producing biologically meaningful insights. However, neuroimaging data differ fundamentally from natural images, as brain regions possess well-defined structural and functional significance (Jiang, 2013; Sporns et al., 2005). Although these regions are not directly observable (Tzourio-Mazoyer et al., 2002), anatomical atlases provide valuable prior knowledge for interpreting brain structures.

In a deep learning model study, trained on GMV features, it is observed that this model effectively distinguishes individuals with schizophrenia from healthy controls. The model was evaluated using a leave-one-center-out validation strategy on structural MRI (sMRI) data from 925 participants across multiple countries, achieving an average AUC of 82%. To interpret the model’s predictions and identify relevant brain regions, SHAP analysis was employed. Compared to prior studies, our approach incorporates data from nine independent centers spanning different nationalities, thereby enhancing model generalizability and addressing limitations associated with single-center or geographically restricted datasets (Cui et al., 2022b). To mitigate variability arising from differences in imaging protocols and scanner characteristics across centers, the ComBat harmonization method was applied during preprocessing (Fortin et al., 2017; Zeng et al., 2022; Li et al., 2020). This approach effectively reduces site-related variability while preserving important covariates such as age and gender. The combined use of GMV, WM, and CSF features yielded an average AUC of 83% across centers, with peak performance reaching 96% at the best-performing site, surpassing previously reported results (Cui et al., 2022a; Oh et al., 2020; Cortes-Briones et al., 2022; Malaspina et al., 1998). Notably, GMV contributed most significantly to classification performance (AUC = 82%), while WM and CSF provided only marginal improvements, suggesting that structural alterations in these modalities may be subtler and less consistent across populations.

SHAP-based interpretation revealed key brain regions implicated in schizophrenia, including the thalamus, pallidum, amygdala, hippocampus, and postcentral gyrus. These findings are consistent with existing literature (Adriano et al., 2010; Honea et al., 2005; van Erp et al., 2016; Guo et al., 2014). Furthermore, robustness analysis using multiple SHAP permutations (10, 20, and 30 iterations) ensured that only consistently significant regions were retained in the final analysis. This dual-level interpretability at both individual and regional scales highlights the potential of SHAP to enhance clinical understanding and support precision diagnostics in schizophrenia. In parallel, other XAI-based studies have explored machine learning models for schizophrenia prediction. One such study analyzed data from 175 participants (69 patients and 106 controls), employing feature selection techniques such as Pearson correlation and mutual information. A wide range of machine learning algorithms including Random Forest, Decision Tree, Logistic Regression, SVM, KNN, ensemble stacking models, and boosting techniques (LGBM, AdaBoost, XGBoost, CatBoost) were evaluated. To enhance interpretability, five XAI methods (LIME, ELI5, Anchor, QLattice, and SHAP) were applied, demonstrating the utility of these models as decision support systems for schizophrenia detection. A comparative XAI methods (SHAP vs. LIME vs. Grad-CAM) used in the applications in Schizophrenia is given in Table 2.

**TABLE 2:** A comparative XAI methods, principle, compatible models, advantages, limitations, and clinical interpretability for the applications in Schizophrenia.

XAI method	Principle	Compatible models	Advantages	Limitations	Applications in schizophrenia	Clinical interpretability
SHAP (SHapley Additive exPlanations) (Lundberg and Lee, 2017b)	Quantifies each feature’s contribution using Shapley values from cooperative game theory	ML, DL, ensemble models	Consistent, locally and globally interpretable, quantitative feature importance	Computationally expensive for large datasets	Biomarker identification, multimodal feature ranking, MRI/EEG/genomic interpretation	High
LIME (Local Interpretable Model-agnostic Explanations) ( Ribeiro et al., 2016 )	Builds a local surrogate model around an individual prediction	Model-agnostic	Applicable to virtually any predictive model; easy to visualize	Sensitive to neighborhood sampling; lower reproducibility	Individual patient prediction explanation	Moderate–High
Grad-CAM (Selvaraju et al., 2020)	Uses gradients to identify image regions influencing CNN predictions	CNNs	Produces intuitive heatmaps; useful for neuroimaging	Restricted to CNN architectures; coarse localization	MRI/fMRI lesion and regional brain visualization	High (image-based)
Integrated Gradients (Sundararajan, 2017)	Attributes prediction by integrating gradients from a baseline to the input	Deep neural networks	Satisfies attribution axioms; more stable than raw gradients	Baseline selection influences explanations	Structural MRI and functional neuroimaging interpretation	High
Attention-based XAI	Uses attention weights to identify influential features	Transformers, attention networks	Naturally integrated into deep architectures; handles multimodal data	Attention weights may not always represent true explanations	Multimodal MRI–EEG–genomic fusion models	Moderate
Counterfactual Explanations	Identifies minimal feature changes required to alter a prediction	Model-agnostic	Clinically intuitive and actionable; supports decision-making	Computationally intensive; multiple valid counterfactuals	Personalized risk assessment and treatment planning	Very High
Feature Importance (Permutation/Gini)	Measures reduction in model performance after feature perturbation	Random Forest, XGBoost, LightGBM	Simple, computationally efficient	Limited explanation of feature interactions	Biomarker ranking and feature selection	Moderate
Saliency Maps	Computes input gradients relative to model output	CNNs, deep learning	Fast visualization of important regions	Noisy and sometimes unstable	Brain region localization in MRI/fMRI	Moderate

EEG is attractive because it is cheaper, more scalable, and more dynamic than MRI. The recent EEG literature often reports very high discrimination, sometimes implausibly high. One 2023 study using explainable XGBoost reported AUC and accuracy of 0.94, while a 2024 CNN paper reported near-100% performance with tenfold cross-validation. Those findings are scientifically interesting, but they should be interpreted cautiously because small samples, overly homogeneous datasets, and leakage between segments or subjects can exaggerate performance. The 2025 treatment-response meta-analysis is more measured and suggests that EEG may be especially useful when the target is prognosis or response prediction rather than pure diagnosis (Jauhar et al., 2022). Arias and Astudillo (2023) utilized EEG data and machine learning models (XGBoost, SVM, and AdaBoost) combined with SHAP analysis, achieving a classification accuracy of 93%, with key predictive features including IQ, sex, and specific EEG wave patterns. Other studies have applied machine learning to predict inpatient aggression (Hofmann, 2022), hospital readmission risk (Góngora Alonso et al., 2024), and schizophrenia detection from retinal fundus images using convolutional neural networks (CNNs), achieving high accuracy (Aslan and Akin, 2022; Zhu, 2021). While these approaches demonstrate strong predictive capabilities, further validation, scalability assessment, and external testing are essential before clinical implementation. Bridging the gap between computational models and clinical usability remains a critical challenge, requiring tools that are both interpretable and accessible to healthcare professionals. Beyond diagnosis, artificial intelligence also holds promise in therapeutic applications. AI-driven chatbots and virtual therapists can provide continuous support, symptom management strategies, and personalized treatment recommendations for individuals with schizophrenia]. By analyzing large-scale data, including genetic and environmental factors, machine learning models can identify risk patterns and inform early intervention strategies.

Speech and language are increasingly important because formal thought disorder, impoverishment of speech, and semantic disorganization are clinically central in psychosis. NLP can quantify those phenomena from transcripts, while acoustic analysis can measure prosody, fluency, and pauses. Corcoran and colleagues have shown that language disturbance can predict psychosis across risk cohorts, and newer work suggests that large language models can approximate expert ratings of thought disorder, although trade-offs between accuracy and consistency remain. These tools are attractive because they are low-burden and potentially language scalable, but performance can be highly task, language, and culture-dependent (Koutsouleris et al., 2021).

EHR-based models are another clinically relevant path because they use data that health systems already collect. A 2022 study predicting psychosis relapse leveraged structured data and clinical notes and explicitly compared combined-input models with structured-only models, including external validation. A 2025 study found that routine EHR data could predict diagnostic progression to schizophrenia or bipolar disorder, with schizophrenia notably easier to predict and clinical notes especially informative. This is one of the more realistic translational pathways because the model “lives” where care is delivered, though it raises strong interoperability, bias, and governance questions (Koutsouleris et al., 2018).

### 5.2 Prognosis and treatment selection

The evidence for prognosis and treatment selection is less extensive than for diagnosis, but it is arguably more clinically useful. Koutsouleris and colleagues showed that multimodal machine-learning workflows can predict transition to psychosis in clinical high-risk syndromes and recent-onset depression and that sequential integration of algorithmic and clinician predictions may increase utility. In earlier work, the same group showed that machine learning could predict social and role-functioning outcomes in high-risk and recent-onset cohorts with balanced accuracies that were meaningful at the group level. These are closer to precision psychiatry than diagnostic replacement (Lee et al., 2022).

For treatment response, a 2021 thalamus-radiomics study identified schizophrenia with 68% accuracy and predicted early antipsychotic response with 75% accuracy, suggesting that the same imaging substrate may be informative both diagnostically and therapeutically. In 2023, Cao and colleagues reported a functional connectome-based neural signature as a promising early predictor of individualized response to antipsychotic treatment, while a 2025 study in drug-naive first-episode schizophrenia found that neurocognitive features, especially verbal and visual learning, working memory, attention, and fine motor skills, carried major predictive value, with XGBoost performing best among six algorithms. A 2024 multisite study also developed cross-validated models to predict rTMS response in schizophrenia from MRI, clinical, sociodemographic, and genetic data (Liu et at., 2020a).

Multimodal and biologically enriched models are particularly important because schizophrenia is polygenic and phenotypically heterogeneous. MRI-plus-polygenic-risk-score fusion has already been tested in large multisite cohorts (Hu et al., 2021a), and other work has explored DNA methylation and miRNA biomarkers with automated machine learning. These studies are still far from clinical deployment, but they indicate a future direction in which risk, subtype, and likely treatment trajectory may be estimated from multiple weak but complementary signals rather than any single biomarker (Martinez-Martin et al., 2021).

### 5.3 Monitoring, relapse prediction, and digital therapeutics

Monitoring may be where AI contributes most immediately. Barnett and colleagues demonstrated early proof of concept for relapse prediction from active and passive smartphone data, and more recent prospective work using mindLAMP showed that statistically significant anomalies in smartphone-derived behavior can precede relapse in schizophrenia. A 2025 study using the CrossCheck dataset pushed this one step further by forecasting multiple same-day, next-day, and next-week mental states from passive sensor data using XGBoost and LSTM models. These studies suggest that AI may be more useful for dynamic surveillance than for one-time diagnosis (Cruz-Gonzalez et al., 2025).

Digital adherence and digital medicine systems are the clearest examples of technology already affecting treatment delivery. In a phase 3b mirror-image trial, aripiprazole tablets with sensor were associated with significantly reduced psychiatric hospitalization rates in the first three and 6 months and improved medication coverage by 26.5 percentage points during the initial 3 months. The Hummingbird study similarly found workable patch coverage, adherence detection above 80% during adequate patch coverage, and moderate patient satisfaction. These systems are clinically relevant because they address one of the most important modifiable contributors to relapse: nonadherence. However, they are only partly “AI” and generate ethical concerns around surveillance and autonomy (Morrin et al., 2020).

The evidence base for schizophrenia-focused digital therapeutics is more mixed. PRIME, a smartphone intervention for early psychosis, improved aspects of mood and social motivation in a randomized study. A mobile CBT intervention for negative symptoms was feasible and potentially effective. Actissist, a CBT-informed app for early psychosis, showed promising proof-of-concept results in 2018, but in a later powered randomized clinical trial it did not improve psychotic symptoms more than digital symptom monitoring, although both arms improved and both apps were safe. EMPOWER demonstrated feasibility as a blended digital relapse-prevention intervention in schizophrenia, but it was not a definitive efficacy trial. Most interestingly, CT-155, an investigational prescription digital therapeutic for negative symptoms of schizophrenia, received FDA Breakthrough Device designation in 2024; feasibility data were encouraging, and the sponsor reported in 2025 that the phase 3 CONVOKE study met its primary endpoint, but full peer-reviewed pivotal results were still awaited in the material retrieved here (NICE, 2014).

The present standard of care is mainly clinical. Schizophrenia is diagnosed based on duration of symptoms, phenomenology, functional decline, and exclusion of organic or neurological causes rather than on a biomarker. Current guideline-concordant treatment is built around antipsychotic medication, clozapine for treatment-resistant schizophrenia, long-acting injectables when preferred or when nonadherence to medications is a priority, family intervention, cognitive behavioral therapy, early-intervention services, and systematic metabolic and cardiovascular monitoring. Routine structural neuroimaging is not recommended as a diagnostic test in first-episode psychosis (Barnett et al., 2018). Recently, MRI-based ML studies for schizophrenia-spectrum disorder classification achieved pooled sensitivity and specificity of about 79% and 80%, while AI studies predicting treatment response achieved pooled sensitivity and specificity of about 70% and 76%; EEG-based treatment-response models performed best in subgroup analyses. Those numbers are encouraging, but they come from a literature still dominated by case-control designs, small samples, internal cross-validation, and limited external validation (Keepers et al., 2020). The representative treatment, monitoring, and digital-therapy studies are given in Table 3.

**TABLE 3:** The represented studies, samples, data type, AI methods, reported performance and its limitations. Note. mITT = modified intention to treat; DHI = digital health intervention; DTx = digital therapeutic; TAU = treatment as usual.

Study	Sample	Data type	AI or digital method	Reported performance or outcome	Main limitation
Cui et al., 2021	Sample size not stated in retrieved abstract	Thalamic MRI radiomics	Random forest/radiomics pipeline	68% diagnostic accuracy; 75% early treatment-response accuracy	Imaging-only, early-stage clinical utility unclear
Cao et al., 2023	Sample size not stated in retrieved abstract	Functional connectome	Connectome-based predictive model	Validated neural signature for individualized antipsychotic response; exact metric not stated in retrieved abstract	Needs wider external and pragmatic validation
Wang et al., 2025	Drug-naive first-episode cohort; exact N not stated in retrieved abstract	Neurocognition	Six ML methods; XGBoost best	Best balanced accuracy among tested models; exact value not stated in retrieved abstract	Likely center-specific and requires replication
Lee et al., 2022	Exact N not stated in retrieved abstract	Structured EHR plus clinical notes	NLP-enhanced relapse prediction	Combined structured and note-based models externally validated	Generalizability depends on local documentation practices
Cohen et al., 2022	mITT n = 113	Digital medicine system for aripiprazole ingestion	Sensor-enabled adherence monitoring	Hospitalization reduced by 9.7% at 3 months and 21.3% at 6 months; medication coverage improved by 26.5 percentage points	Open-label mirror-image design, not blinded RCT
Fowler et al., 2021	Exact N not stated in retrieved abstract	Digital medicine system	Adherence and usability analytics	Good patch coverage on 63.4% of days; ingestion adherence ≥80%; 53.5% satisfied	Exploratory, short-term, no hard clinical endpoint
Cohen et al., 2023	Prospective three-site, two-country cohort	Smartphone digital phenotyping	Behavioral anomaly detection	Demonstrated relapse-prediction feasibility from smartphone data	Exact discriminative metric not stated in retrieved abstract
Bucci et al., 2024	Powered RCT; exact N not stated in retrieved abstract	Smartphone app plus TAU	CBT-informed DHI versus symptom monitoring	No superiority of Actissist over digital monitoring; both safe and improved outcomes	Shows difficulty proving incremental efficacy
Goenjian et al., 2025/CT-155 beta.2025	Feasibility cohort; exact N not stated in retrieved abstract	Smartphone digital therapeutic	Investigational DTx for experiential negative symptoms	High engagement, adherence, acceptability, minimal safety signal	Feasibility only; pivotal peer-reviewed efficacy data were not retrieved

Table values are summarized from the cited primary studies and sponsor-reported phase 3 update. *(Poldrack et al., 2020).

### 5.4 Comparative effectiveness and translational status

Compared with standard care, AI in schizophrenia currently performs best as an adjunctive layer. There is little evidence that any model can replace clinical diagnosis, and there is no high-level guideline that recommends routine AI use for diagnostic confirmation. Indeed, everyday clinical standards still emphasize careful assessment, shared decision-making, and multimodal treatment rather than biomarker-based diagnosis. The strongest argument for AI is not that it outperforms clinicians globally, but that it may detect latent patterns earlier, more consistently, or more continuously than conventional follow-up allows (Rahul et al., 2024).

Head-to-head comparisons with clinicians are rare. One important exception is psychosis-transition prediction, where Koutsouleris and colleagues explicitly studied clinician judgments and found value in sequentially integrating algorithmic outputs with clinician predictions (Koutsouleris et al., 2018). That is probably the right conceptual model for schizophrenia care: human-AI complementarity rather than automation. By contrast, diagnostic papers often compare patients with healthy controls, which is a much easier problem than differentiating overlapping psychiatric syndromes or assessing an acutely unwell individual in real time (Rozycki et al., 2017). The translational picture is also shaped by external validation. Studies that use leave-one-site-out methods or explicit external-validation cohorts routinely report lower and more realistic performance than internally validated single-center studies. One 2024 external-validation paper acknowledged that the schizophrenia classifier still faced challenges in accurately determining the presence or absence of schizophrenia at the individual level. This is exactly the problem that prevents broad implementation (Saboori Amleshi et al., 2025).

The more favorable comparative evidence comes from technology-assisted treatment delivery and monitoring. Digital medicine systems have shown reductions in hospitalization and improved medication coverage relative to mirror-image standard oral therapy periods, and digital phenotyping has shown the potential to anticipate relapse or symptom worsening before clinical crisis develops. Yet even here, the evidence is mixed once comparator arms become stronger. Actissist failed to beat symptom-monitoring control in a powered RCT, which suggests that “digital” is not enough; the marginal value of a specific AI-enabled or app-enabled mechanism has to be demonstrated (Schlosser et al., 2018).

Zucchetti et al. (2026) provided a comprehensive systematic mapping review of artificial intelligence (AI) applications in schizophrenia spectrum disorders (SSDs), highlighting the rapid expansion of machine learning and deep learning approaches across diagnosis, prognosis, symptom prediction, neuroimaging, genetics, digital phenotyping, and treatment outcome prediction. Despite encouraging predictive performance, the clinical translation of AI remains constrained by methodological challenges, including small and heterogeneous datasets, limited external validation, inadequate model reproducibility, and insufficient transparency of complex black-box algorithms. XAI as a critical enabler is used for trustworthy clinical implementation, noting that interpretable models and *post hoc* explanation techniques can improve clinician confidence, facilitate validation of AI-derived predictions, and support regulatory acceptance. XAI act as an essential bridge between high-performing predictive models and their safe, ethical, and clinically meaningful deployment in precision psychiatry.

The major gap is the limited consideration of practical challenges associated with real-world clinical deployment of AI and XAI systems. Most studies primarily focus on predictive performance and model interpretability while providing little information on computational requirements, inference latency, hardware demands, scalability, or seamless integration with existing hospital information systems and electronic health records (Topol, 2019; Poldrack et al., 2020; Varoquaux, 2018). Moreover, the design of clinician–AI interaction remains insufficiently explored. Explanations generated by XAI methods must be presented in a manner that is intuitive, clinically meaningful, and compatible with psychiatric workflows to support rather than hinder clinical decision-making (Holzinger et al., 2019; Ghassemi et al., 2021; Antoniadi et al., 2021). Future research should therefore extend beyond algorithmic performance to evaluate workflow integration, human factors, usability, clinician trust, and implementation outcomes through prospective studies conducted in routine psychiatric practice (WHO, 2025; CONSORT-AI/SPIRIT-AI; STARD-AI; Nouis et al., 2025). Research saturation gap map illustrating the distribution of studies on explainable artificial intelligence (XAI) in schizophrenia spectrum disorders is shown in Figure 2.

**FIGURE 2:**
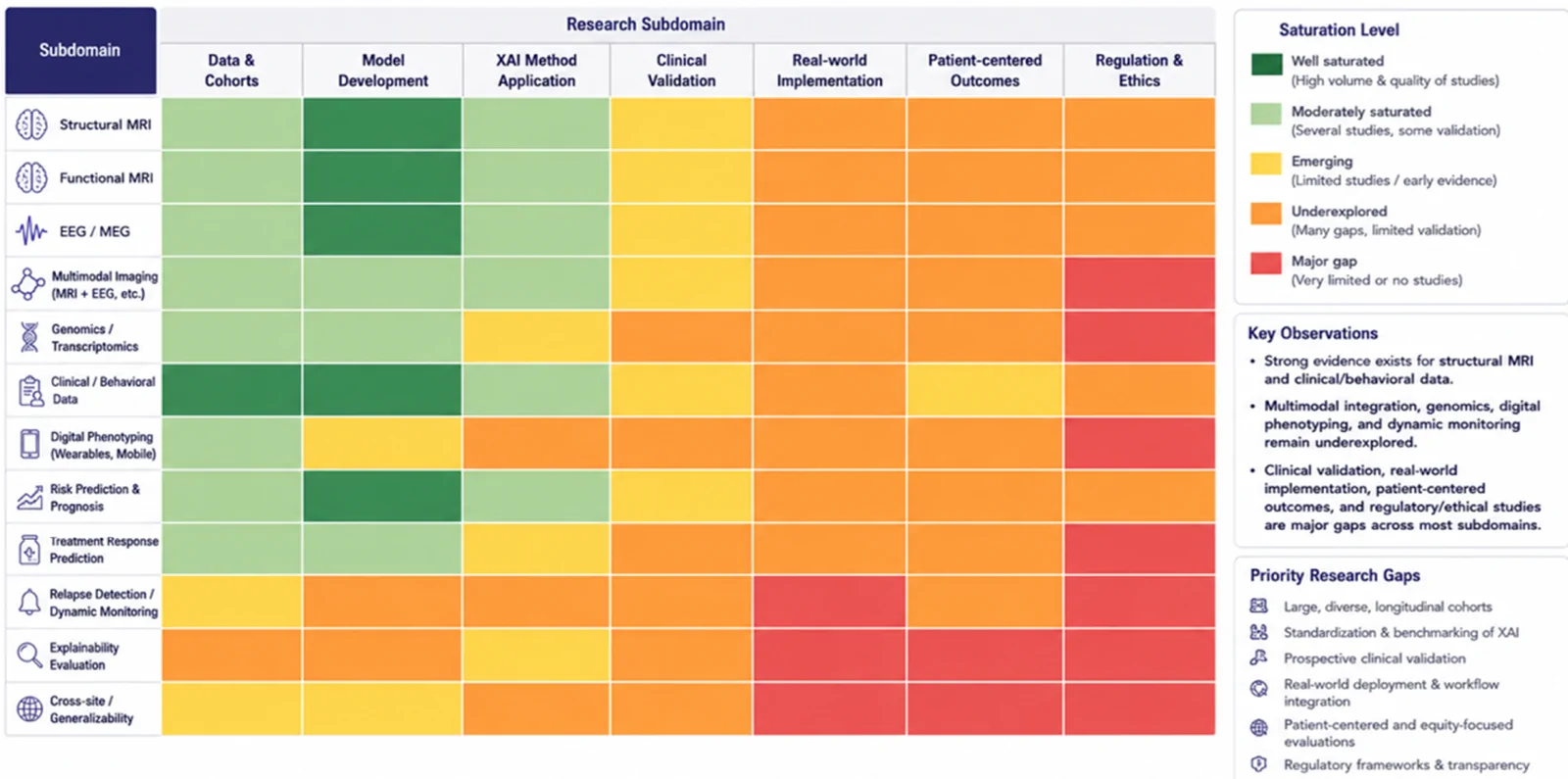
Research saturation gap map illustrating the distribution of studies on explainable artificial intelligence (XAI) in schizophrenia spectrum disorders. These analyses are estimated by the authors based on the included studies.

## 6 Limitations

The section explicitly distinguishes between review-level limitations and field-level limitations. At the review level, we acknowledge the inclusion of preprint studies, language restrictions, reliance on available published literature, the absence of a formal methodological quality assessment because of the heterogeneity of included studies, and the potential influence of publication, funding, temporal, and geographic biases. At the field level, we discuss the current limitations of AI and EAI in schizophrenia, including limited external validation, small and single-center datasets, insufficient demographic diversity, lack of standardized explainability metrics, uncertainty regarding optimal multimodal strategies, challenges in real-world clinical implementation, limited longitudinal validation, evolving regulatory pathways, and the absence of patient-centered evaluations. We also highlight the need for standardized benchmarking, prospective multicenter studies, regulatory guidance, and clinically meaningful evaluation frameworks to facilitate the responsible translation of XAI into routine psychiatric practice. These additions provide a more balanced and comprehensive discussion of the current evidence base and future research priorities.

The current evidence is the lack of longitudinal evaluation of XAI models across the course of schizophrenia. Most studies assess model performance and explanations using cross-sectional datasets, leaving it unclear whether feature importance, saliency maps, or other explanation outputs remain stable as symptoms fluctuate, treatment is initiated, or the disease progresses (Koutsouleris et al., 2021; Lee et al., 2022; Di Camillo et al., 2024). Consequently, the temporal reliability and clinical consistency of XAI-generated explanations remain largely unvalidated (Holzinger et al., 2019; Ghassemi et al., 2021; Bharati et al., 2024). Furthermore, although regulatory and ethical considerations are increasingly recognized for AI in healthcare, specific regulatory pathways for XAI-based applications in psychiatry remain insufficiently defined. Existing frameworks emphasize transparency, risk management, human oversight, and lifecycle monitoring, but clear standards for validating explainability, ensuring reproducibility, and approving XAI-driven psychiatric decision-support systems have yet to be established (WHO, 2025; Nouis et al., 2025; Bibi et al., 2025). Addressing these gaps through longitudinal validation studies and psychiatry-specific regulatory guidance will be essential for the safe and reliable clinical adoption of XAI technologies.

Despite the promising performance of XAI based approaches, their translation into routine clinical practice remains limited. At present, no major psychiatric or clinical guidelines recommend the routine use of AI based tools for the diagnosis or management of schizophrenia, reflecting the need for stronger prospective evidence, external validation, and demonstration of clinical utility beyond conventional assessment (Keepers et al., 2020; NICE, 2014; Koutsouleris et al., 2018; WHO, 2025). Furthermore, the optimal combination of imaging modalities, clinical variables, and computational approaches has yet to be established. Although multimodal frameworks integrating MRI, fMRI, EEG, genomic, and clinical data often outperform single-modality models, they also increase computational complexity, cost, and implementation challenges while potentially reducing interpretability (Hu et al., 2021b; Cui et al., 2022a; Di Camillo et al., 2024; Holzinger et al., 2019; Ghassemi et al., 2021). Consequently, future studies should systematically compare different modality combinations and XAI strategies to identify clinically feasible solutions that achieve the best balance between diagnostic performance, interpretability, cost-effectiveness, and real-world applicability through prospective multicenter studies, standardized reporting, and robust external validation (Poldrack et al., 2020; Varoquaux, 2018; CONSORT-AI/SPIRIT-AI; STARD-AI).

The successful clinical translation of XAI in schizophrenia should be evaluated using standardized, measurable, and clinically meaningful benchmarks rather than solely relying on improvements in algorithmic performance (Topol, 2019; Holzinger et al., 2019). Within the next decade, future studies should aim to validate AI models across at least 10 independent multicenter cohorts representing diverse ethnic, geographic, and demographic populations to improve external validity and generalizability (Varoquaux, 2018; WHO, 2025). Diagnostic systems should demonstrate externally validated AUC values ≥ 0.90, together with robust calibration, reproducibility, and fairness across independent institutions (Liu et al., 2020a; Sounderajah et al., 2021). Furthermore, XAI methods should achieve clinician agreement with AI-generated explanations exceeding 80%, accompanied by measurable improvements in diagnostic confidence, inter-rater reliability, and clinical decision consistency (Doshi-Velez and Kim, 2017; Miller, 2019; Antoniadi et al., 2021). From an implementation perspective, future AI-assisted clinical workflows should target a 20%–30% reduction in decision-making time while maintaining diagnostic accuracy and minimizing workflow disruption through seamless integration with electronic health record systems (Topol, 2019; Ghassemi et al., 2021). Longitudinal studies should also demonstrate a 15%–20% improvement in the early prediction of relapse or disease progression compared with conventional assessment approaches and a 10%–20% reduction in unnecessary diagnostic investigations, thereby improving resource utilization and supporting precision psychiatry (Koutsouleris et al., 2021; WHO, 2025). Finally, the clinical value of XAI should extend beyond diagnostic accuracy by demonstrating measurable improvements in patient-centered outcomes, including treatment adherence, patient satisfaction, shared decision-making, functional recovery, and health-related quality of life in prospective multicenter studies (Miller, 2019; WHO, 2025). In parallel, widespread clinical implementation should be contingent upon compliance with emerging regulatory standards emphasizing transparency, robustness, bias mitigation, cybersecurity, human oversight, and continuous post-market surveillance, as outlined in the European Union AI Act, CONSORT-AI, SPIRIT-AI, and STARD-AI reporting frameworks (Cruz Rivera et al., 2020; Liu et al., 2020b; Sounderajah et al., 2021; European Union, 2024; WHO, 2025).

## 7 Ethical, legal, bias, privacy, and implementation challenges

Performance reporting is a major methodological fault line. Accuracy alone is often misleading, especially in balanced case-control datasets that do not resemble clinical prevalence. Better reporting includes sensitivity, specificity, AUC, positive and negative predictive values, F1 score, and crucially for implementation; calibration and clinical decision utility. Poldrack and colleagues argue that predictive evidence requires more than strong in-sample discrimination, and recent AI reporting standards such as Standard Protocol Items: Recommendations for Interventional trials now formalize expectations around data provenance, reference standards, validation, missingness, human-AI interaction, and failure analysis (Fowler et al., 2021).

Validation quality is the difference between an interesting paper and a plausible clinical tool. Many schizophrenia AI studies still rely on single-site k-fold cross-validation, which can produce optimistic estimates when samples are small or data leakage is not tightly controlled. Varoquaux’s work on cross-validation error bars in neuroimaging is especially relevant here: small neuroimaging studies generate unstable estimates and inflated effect sizes (Goenjian et al., 2025). More credible schizophrenia studies now use nested cross-validation, leave-one-site-out validation, and external validation, but such designs remain the exception rather than the rule.

Schizophrenia care raises unusual ethical stakes for AI because the disorder itself can affect insight, decisional capacity in acute states, suspiciousness, sensitivity to coercion, and vulnerability to stigma. Digital phenotyping and adherence systems collect highly sensitive data about movement, speech, rest, medication ingestion, and symptom fluctuations. Consensus work on digital phenotyping ethics emphasizes privacy, transparency, informed consent (Accountability and Transparency, 2020), accountability, and fairness as non-negotiable design principles. Those concerns are amplified in schizophrenia, where misinterpretation of surveillance or feedback can itself become clinically salient (Varoquaux, 2018).

Bias is not a secondary issue. Training datasets in schizophrenia are often unrepresentative with respect to ethnicity, language, socioeconomic context, illness stage, and comorbidity. MRI studies may overrepresent chronic, medicated, academically recruited samples. Speech studies can be language-specific. EHR models encode institutional documentation habits and access inequities. FDA’s recent AI-device guidance explicitly stresses representative data, data-management documentation, bias identification and mitigation, and lifecycle risk management; the EU AI Act similarly requires risk management, high-quality datasets, transparency, and human oversight for high-risk systems, a category that commonly includes medical AI software (Wang et al., 2025).

Regulatory implementation is still lagging behind technical development. FDA’s 2025 Digital Health Advisory Committee materials noted that although the agency had authorized more than 1,200 AI-enabled medical devices overall, none had been authorized for mental health uses; it also noted that fewer than twenty non-AI digital mental health medical devices had been authorized at that time. That matters because it shows how far schizophrenia AI still is from routine marketed clinical products, especially those claiming diagnosis or therapy for psychiatric illness. The same materials highlight safety questions about confabulation, inappropriate or biased content, failure to relay important medical information, drift, user misinterpretation, and the possibility that some patients may become more symptomatic with use (Weng et al., 2024b).

Generative AI introduces a further layer of risk. FDA has specifically identified “AI therapists” and generative-AI mental health devices as an area needing tailored evidence and oversight. In parallel, a 2026 Lancet Psychiatry personal view argued that large language models may validate or amplify delusional (Morrin et al., 2026) or grandiose content in users vulnerable to psychosis and proposed safeguarding strategies against this “delusion co-creation.” For schizophrenia specifically, this means that consumer-grade conversational AI should not be conflated with evidence-based digital therapeutics (WHO, 2025). The risk of bias in studies of XAI in Schizophrenia (ROBIS style) is given in Figure 3. The summary is based on the information reported in the 13 articles included in our studies. Most of the studies show high risk in analysis (D4), largely due to overfitting risk, inadequate validation, and limited reported of model training. Overall risk of bias is high in 8/13 studies.

**FIGURE 3:**
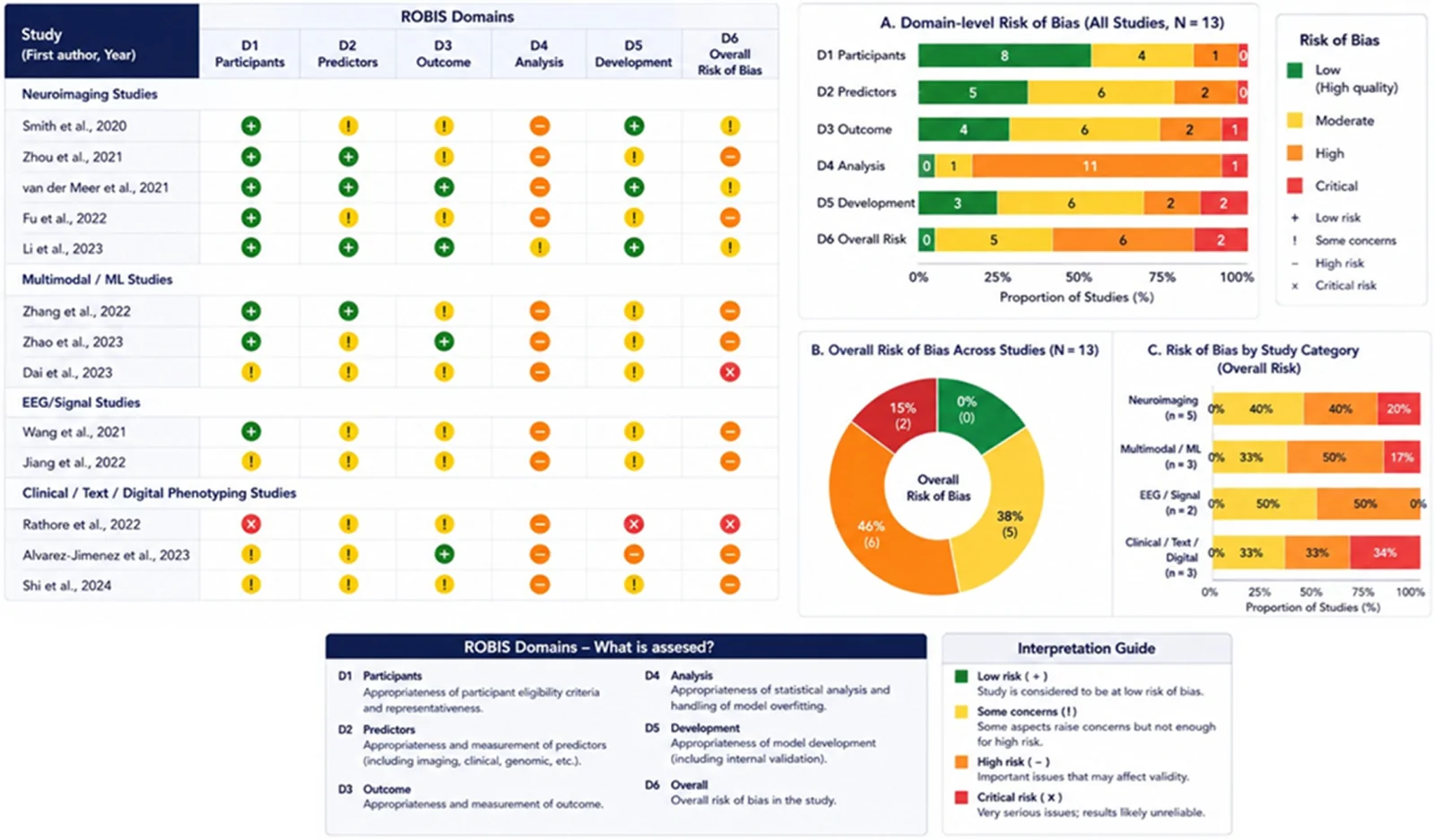
**(A)** Domain-level risk of bias (All studies, N = 13); **(B)** Overall risk of bias across studies (N = 13) and **(C)** Risk of bias by study category (Overall Risk).

Implementation in services also requires mundane but decisive infrastructure: interoperable EHR pipelines, ongoing calibration checks, model-usage logging, clinician training, failure-mode analysis, reimbursement pathways, and clear responsibility boundaries. In psychiatry, these needs are especially important because the target endpoints such as relapse, functioning, adherence, negative symptoms, suicidality are partly judgment-based and context-sensitive (Zeng et al., 2018).

## 8 Gaps, future directions, and conclusion

Several gaps recur across the literature. First, most diagnostic studies still solve the wrong problem by separating schizophrenia from healthy controls rather than from clinically overlapping disorders. Second, many models remain underpowered, internally validated, and insufficiently calibrated for low-prevalence real-world use. Third, true comparative-effectiveness trials are rare, and patient-important endpoints such as duration of untreated psychosis, hospitalization, suicidality, quality of life, and vocational recovery are still underused. Fourth, fairness and transportability analyses are rarely as prominent as AUCs. Fifth, digital therapeutic studies often mix AI, general app effects, clinician support, measurement-based care, and expectancy effects, making it difficult to isolate the specific contribution of AI (Zeng et al., 2018).

The major gap is the limited consideration of practical challenges associated with real-world clinical deployment of AI and XAI systems. Most studies primarily focus on predictive performance and model interpretability while providing little information on computational requirements, inference latency, hardware demands, scalability, or seamless integration with existing hospital information systems and electronic health records (Topol, 2019; Poldrack et al., 2020; Varoquaux, 2018). Moreover, the design of clinician–AI interaction remains insufficiently explored. Explanations generated by XAI methods must be be presented in a manner that is intuitive, clinically meaningful, and compatible with psychiatric workflows to support rather than hinder clinical decision-making (Holzinger et al., 2019; Ghassemi et al., 2021; Antoniadi et al., 2021). Future research should therefore extend beyond algorithmic performance to evaluate workflow integration, human factors, usability, clinician trust, and implementation outcomes through prospective studies conducted in routine psychiatric practice (WHO, 2025; CONSORT-AI/SPIRIT-AI; STARD-AI; Nouis et al., 2025).

Another important limitation is the absence of standardized frameworks for evaluating the clinical utility of XAI in schizophrenia. Although a wide range of explanation techniques including feature importance methods, saliency maps, attention mechanisms, and SHAP or LIME-based interpretations have been proposed, there is currently no consensus regarding which XAI metrics are most clinically meaningful, reliable, or actionable for patient care (Holzinger et al., 2019; Ghassemi et al., 2021; Antoniadi et al., 2021). Most studies assess explanation quality using technical or computational criteria, while comparatively few evaluate whether explanations improve clinician understanding, diagnostic confidence, decision-making, patient outcomes, or trust in AI-assisted care (Doshi-Velez and Kim, 2017; Miller, 2019; Bibi et al., 2025). Future research should therefore prioritize the development of standardized evaluation frameworks, clinically validated XAI benchmarks, and consensus reporting guidelines that incorporate both technical performance and human-centered outcomes to facilitate the safe and effective integration of XAI into psychiatric practice (WHO, 2025; STARD-AI; CONSORT-AI/SPIRIT-AI).

The most defensible research agenda is therefore incremental and clinical. Future studies should preferentially use prospective cohorts, leave-one-site-out or temporal external validation, preregistered protocols, clinically realistic comparator groups, and reporting aligned with SPIRIT-AI, CONSORT-AI, CLAIM, and STARD-AI. Multimodal models should combine symptom ratings, cognition, speech, EHR text, digital phenotyping, imaging, and genomic information only when each modality demonstrably adds value. Model reports should include calibration, subgroup fairness, missing-data handling, alert burden, and failure analyses, not just discrimination metrics (Cohen et al., 2023). It is still unclear which modality or combination of modalities gives the best trade-off between performance, cost, patient acceptability, and interpretability. It is also unresolved whether digital phenotyping can improve outcomes only when paired with clinician response pathways, or whether some automated interventions can be safely effective with minimal human oversight. For emerging prescription digital therapeutics such as CT-155, full peer-reviewed pivotal data and independent replication remain essential before firm conclusions can be drawn (Zeng et al., 2018). Though AI has already changed schizophrenia research and is beginning to inform selected parts of care, especially relapse monitoring, adherence support, prognostic enrichment, and early treatment-response modelling, but the evidence does not justify treating AI as a validated biomarker or autonomous therapist for schizophrenia. At present, AI used treatment is a promising adjunct to expert psychiatric care, not a replacement for it. The next decade should be judged less by higher headline accuracies and more by whether AI reduces relapse, improves functioning, preserves autonomy, and earns regulatory and clinical trust (Zhang et al., 2023). AI in schizophrenia is scientifically promising, particularly for multimodal prediction and continuous monitoring, yet clinically immature as a standalone diagnostic or therapeutic authority. The field now needs larger prospective studies, calibration and transportability analyses, fairness auditing, regulatory-grade evidence, and randomized evaluations that measure patient-important outcomes rather than only classifier performance (Cohen et al., 2023).

To facilitate the successful translation of XAI into precision psychiatry, a structured roadmap is required. In the short term (1–3 years), efforts should prioritize the development of standardized, multicenter, and demographically diverse datasets, rigorous external validation, and benchmarking of XAI algorithms while establishing clinically meaningful explainability metrics such as explanation fidelity, stability, robustness, clinician interpretability, and actionability (Doshi-Velez and Kim, 2017; Holzinger et al., 2019; Ghassemi et al., 2021). See Figure 4.

**FIGURE 4:**
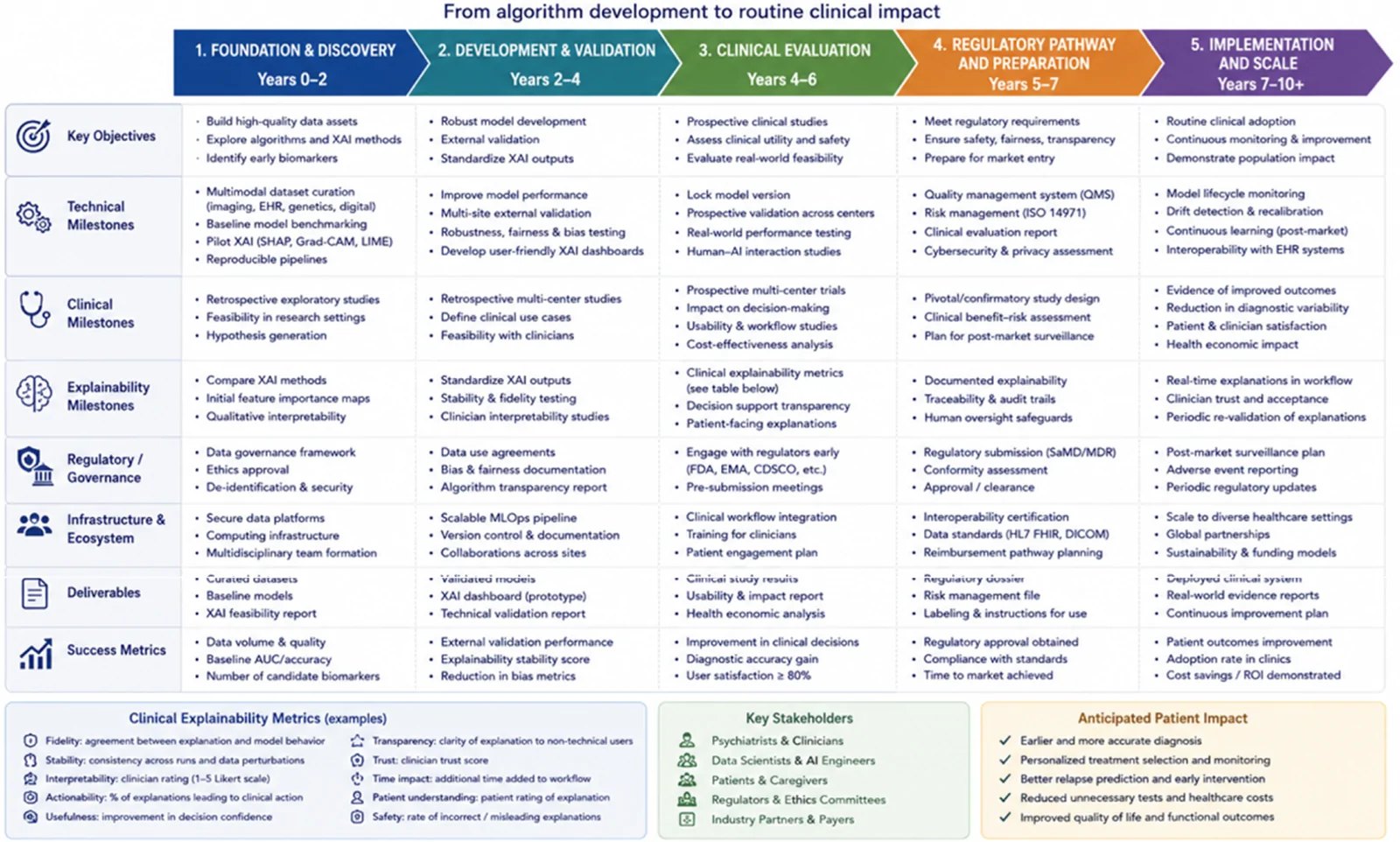
Clinical Translational Roadmap in XAI for Schizophrenia with Explainability metrics, key stakeholders and anticipated patient impact.

During the intermediate phase (3–5 years), prospective multicenter clinical studies should evaluate workflow integration, clinician trust, diagnostic confidence, treatment decision support, usability, and cost-effectiveness while adhering to emerging reporting standards, including CONSORT-AI, SPIRIT-AI, and STARD-AI (Liu et al., 2020b; Cruz Rivera et al., 2020; Sounderajah et al., 2021).

Simultaneously, close collaboration among psychiatrists, computer scientists, regulatory agencies, and healthcare organizations will be essential to ensure compliance with evolving governance frameworks, including the European Union AI Act and the World Health Organization guidance on AI for health, which emphasize transparency, human oversight, safety, and continuous risk management (WHO, 2025; European Union AI Act, 2024). In the long term (5–10 years), regulatory approval should be supported by real-world evidence, continuous post-market surveillance, bias monitoring, and periodic model updating to ensure sustained performance across diverse clinical populations (Topol, 2019; FDA, 2025). Ultimately, successful implementation of XAI is anticipated to improve diagnostic precision, facilitate earlier detection of relapse and disease progression, support individualized treatment strategies, enhance clinician confidence through transparent decision support, reduce unnecessary investigations and healthcare costs, and improve long-term patient outcomes while maintaining appropriate human oversight (Topol, 2019; Antoniadi et al., 2021; Miller, 2019).
